# The efficacy of oral pain relief cocktail during pan-retinal photocoagulation for diabetic retinopathy: a randomized clinical trial

**DOI:** 10.1186/s40942-022-00438-5

**Published:** 2023-02-02

**Authors:** Mohammadkarim Johari, Sarah Safniyat, Mohammadreza Badie, Abdulrahim Amini, Fatemeh Sanie-Jahromi

**Affiliations:** 1 Poostchi Ophthalmology Research Center, Department of Ophthalmology, School of Medicine, Shiraz University of Medical Sciences, Shiraz, Iran; 2 Department of Ophthalmology, School of Medicine, Hormozgan University of medical sciences, Bandar Abbas, Iran

**Keywords:** Diabetic retinopathy, Acetaminophen, Ibuprofen, Caffeine, Pregabalin, Visual analog scale, Pan-retinal photocoagulation

## Abstract

**Purpose:**

to evaluate the pain-relieving effect of analgesic combinations during pan-retinal photocoagulation (PRP) in patients with non-proliferative diabetic retinopathy (NPDR).

**Methods:**

This study was a randomized, double-blind, placebo-controlled trial. Patients with severe NPDR without previous history of PRP were included in the study. Both eyes of the patients were treated with a pan-retinal photocoagulation procedure. The retina was divided into four quadrants and the treatment plan for patients submitted to PRP was divided into four sessions. Different oral medications were given to patients 1 hour before the procedure. Capsules containing a combination of analgesic drugs (including 325 mg acetaminophen, 200 mg ibuprofen, and 40 mg caffeine, referred to as N), pregabalin capsules (75 mg, referred to as P), a combination of N capsules and P capsules (referred to as NP), and the placebo were used in each session. Each patient scored the pain sensation immediately after the procedure using a visual analog scale (VAS).

**Result:**

60 eyes of 30 patients were studied. The mean value of VAS in patients receiving the placebo was 3.3 ± 1.822 units, while this scale was 3.067 ± 1.507, 3.5 ± 1.479, and 3.5 ± 1.77 in the N, P, and NP consumed patients, respectively. There was no significant difference in VAS levels and the patient’s vital signs between different sessions (P = 0.512).

**Conclusion:**

No evidence of the pain-relieving effect of N, P or NP was found during PRP.

*Trial registration*: IRCT20200915048724N1. Registered 20 October 2020, https://www.irct.ir/trial/51345

## Introduction

Diabetic retinopathy (DR) is a microvascular disorder that occurs due to the long-term effects of diabetes. It leads to threatening visual damage to the retina and, eventually, blindness. DR is the most common cause of vision loss in adult patients of working age in the world [1]. Early diagnosis and timely intervention are required to prevent blindness. The structural injuries to the retinal blood vessels in response to the metabolic changes in diabetic patients, as well as ischemic or hypoxia conditions in the retina lead to the activation of vascular endothelial growth factor (VEGF) and hence exacerbates diabetic retinal angiogenesis. VEGF is believed to be a critical factor in the progression of DR, and therefore anti-angiogenesis drugs have emerged as the pioneers in the DR treatment [2, 3]. Laser photocoagulation has been the gold standard for the treatment of diabetic macular edema (DME), and proliferative diabetic retinopathy (PDR) before the advent of anti-VEGF therapy [4]. Pan-retinal photocoagulation (PRP) has also been used to treat PDR and significantly reduces the risk of severe vision loss, especially in cases with high-risk complications such as vitreous hemorrhage [5]. However, previous studies have shown that PRP is painful for most patients. Therefore, pain relief strategies during PRP are necessary to reduce the patient’s suffering [6, 7]. There are several options for lowering PRP-related pain on an outpatient basis. Retrobulbar anesthesia, peribulbar anesthesia, and sub-tenon anesthesia are effective methods for relieving pain. However, these methods include invasive procedures that might be accompanied by potential complications [8]. Oral or intramuscular routes of anesthetic drug delivery to the retina are safe and associated with no ocular complications [9]. Acetaminophen, ibuprofen, caffeine, and pregabalin are anti-inflammatory analgesics. The drugs are widely used for pain relief. To our knowledge, there are no reports on the use of combination of these drugs for pain relief of DR patients during the PRP procedure. This clinical trial study aimed to investigate the analgesic effect of oral administration these drugs as a pretreatment for diabetic patients under PRP.

## Methods

### Study design, patient selection and clinical procedure

This study was a randomized, double-blind, placebo-controlled trial (IRCT20200915048724N1) conducted at Shiraz University of Medical Sciences, Shiraz, Iran. Participants were recruited between April 2021 and April 2022 from Motahari Clinic, Shiraz, Iran. Eligible participants were informed about the study procedures. All patients gave their informed written consent to participate in the study and thoroughly explained once the risks and benefits of the intervention. This study was approved by the Human Ethics Committee of the Shiraz University of Medical Science (IR.SUMS.MED.REC.1399.360, IRCT registration number: IRCT20200915048724N1, Registration date: 29/10/2020). This study followed the CONSORT statement.

Patients with severe non-proliferative diabetic retinopathy (NPDR) without previous history of PRP were included in the study.

Patients with a history of hypersensitivity or contraindication to acetaminophen, nonsteroidal anti-inflammatory drug (NSAIDs), and pregabalin, previous chronic eye pain such as glaucoma and dry eye, previous PRP, current use of painkillers for any other disease, media opacity or vitreous hemorrhage were excluded from the study.

**Fig. 1:**
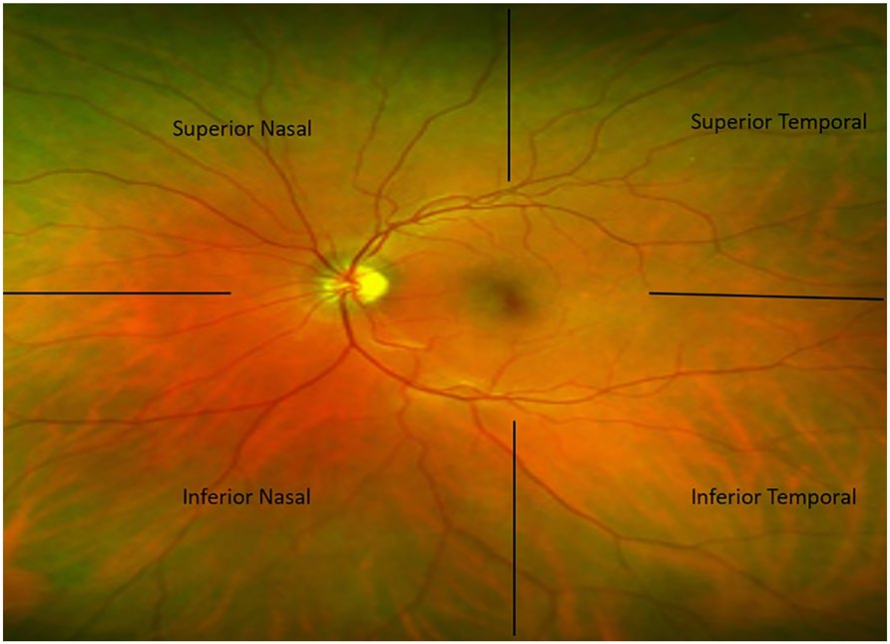
The retina was divided into four quadrants (Superior temporal/Superior nasal/Inferior temporal/Inferior nasal) with the center of the macula, in each session, the same area of both retinas underwent laser therapy

In this factorial-designed study, both eyes of the patients were treated with a pan-retinal photocoagulation procedure. The treatment plan for patients submitted to PRP was divided into four sessions. The retina was divided into four quadrants, namely inferior nasal (IN), inferior temporal (IT), superior nasal (SN), and superior temporal (ST), with the center of the macula (Fig. 1). The same area of both retinas underwent PRP (first session; IN of both eyes, followed by IT, SN, and ST in each session). Different pre-procedure medications were used to reduce the patient’s pain during the PRP in each session. Four treatment regimens were designed; capsules containing a combination of analgesic drugs (including 325 mg acetaminophen, 200 mg ibuprofen, and 40 mg caffeine, referred to as N in this study; Razavi Company, Mashhad, Iran), pregabalin capsules (75 mg, referred to as P in this study; Jalinos Company), a combination of N and P capsules (referred to as NP), and the placebo. The assistant who had no role in treating the patients sent randomization tables and allocated each pill in a randomized sequence. Tablets were packaged in a similar way and were given to patients 1 hour before the procedure. Patients and investigators were blind about the type of medications.

PRP procedure was performed by a single retinal specialist (MKJ). The pupils were dilated using 1% tropicamide. Thirty minutes later, we used a drop of proparacaine hydrochloride 0.5% to each eye for corneal anesthesia.

Laser treatment was performed with a pattern of 532 nm frequency-diode laser (Integre Pro Scan™, Ellex medical company, USA) using a SuperQuad 160 contact lens (laser spot magnification of 2.0; Volk Optical Inc., Mentor, OH). Each session consisted of approximately 500 spots, with laser energy adjusted to achieve moderate white burns, the spots size of 500 micrometers, and an exposure time of 0.15 s; each session lasted between 5 and 10 min for both eyes (Table 1), in all quadrants visible ciliary nerves were spared from burning by laser spots. To clear all the effects of the previous session’s drugs, the interval between sessions was adjusted to 2 weeks. The areas were treated in the following order: IN, IT, SN, ST.

**Table 1:** Comparison of laser parameters and VAS regarding to different retinal quadrants

Retinal quadrants	Mean Laser Power (Milliwatt)	Mean Laser spot number (Sum of both eye)	Mean Laser spot size (µm)	Mean VAS score (µm)
SN	400 ± 50	510 ± 54	500	3.1 ± 2.7
IN	395 ± 59	512 ± 27	500	3.2 ± 1.3
ST	410 ± 65	528 ± 32	500	3.1 ± 3.9
IT	409 ± 45	532 ± 14	500	3.3 ± 6.1

*VAS* visual analog scale, *IT* Inferior Temporal, *IN* Inferior nasal, *ST* Superior temporal, *SN* Superior nasal

Before the laser treatment sessions, each patient underwent a complete ophthalmological examination utilizing a slit lamp, indirect ophthalmoscopy and Goldmann applanation tonometry. The blood pressure and heart rate were recorded with a digital monitor before and immediately after the laser treatment.

Following each laser session, immediately, the patients were asked to provide feedback on the degree of pain experienced. The method chosen was a numerical and facial expression pain scale adapted from a visual analog scale (VAS), consisting of a 10 cm scale labeled with numbers from 0 to 10 and a face pain rating scale with a number from 0-2-4-6-8-10, which was used to determine the intensity of the pain. The blinded examiner instructed patients that 0 represents experiencing no pain and ten means experiencing the maximum pain they could imagine, an excellent clinical pain indicator of pain intensity in postoperative patients. The blinded examiner evaluated the patient’s facial expression (objective score) and asked patients to score a number between 0 and 10 regarding their pain experience during the procedure (subjective score); the mean of both numbers was recorded for each session.

### Statistical analysis

All data were processed with SPSS version 20.0. T-test, one-way ANOVA, and EP16 were applied for statistical analyses. Categorical data were analyzed using a chi-squared test (χ2) and reported as Mean ± SD. A P-value of less than 0.05 was considered statistically significant.

## Results

**Fig. 2:**
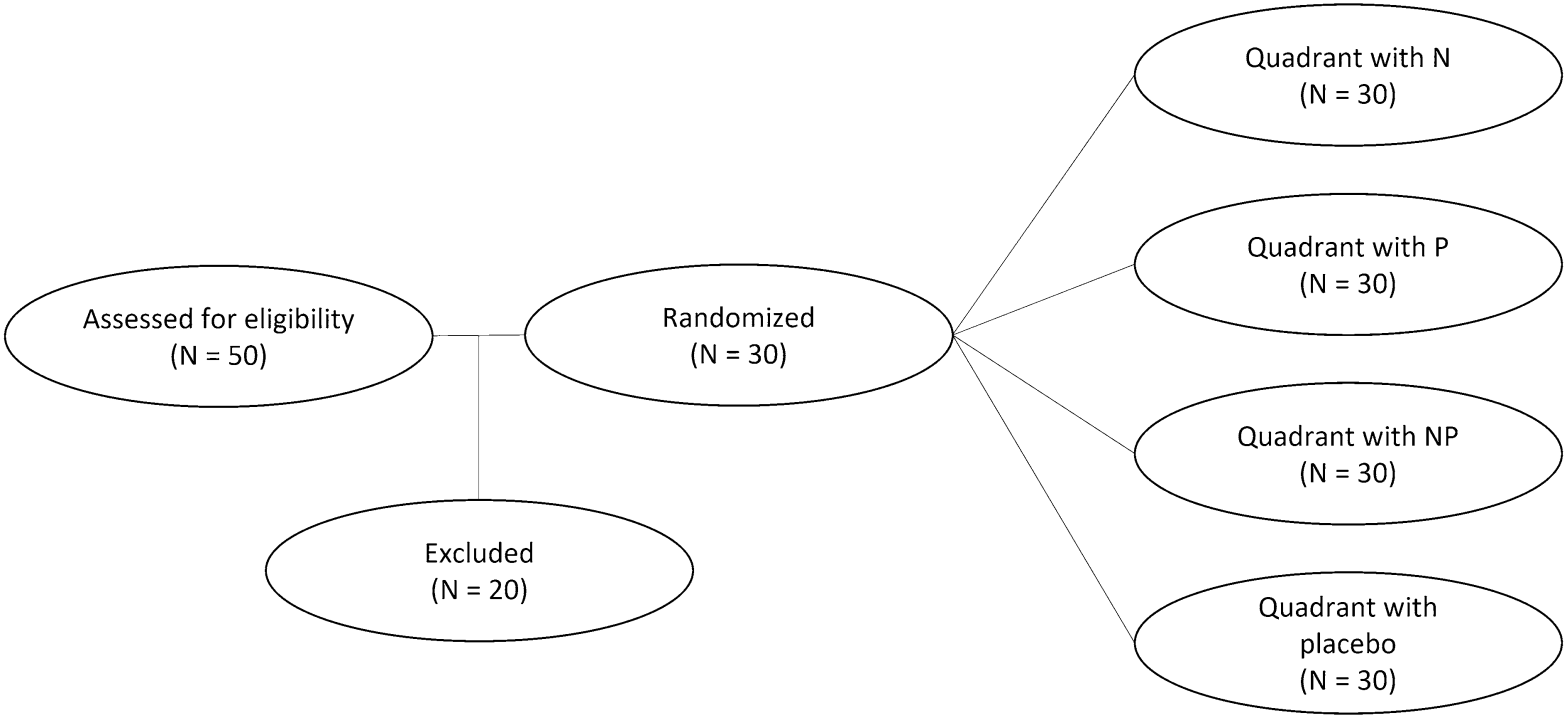
Consort flow diagram

In the present study, a total of 50 patients (100 eyes) receiving the first session of PRP treatment were enrolled. Ten patients discontinued the study because of difficulty adhering to third and fourth laser sessions due to COVID-19 respiratory symptoms and quarantine. Seven were removed due to taking other analgesic drugs between sessions due to musculoskeletal pain, and three were discontinued due to vitreous opacity or hemorrhage. Finally, 30 patients (60 eyes) were analyzed (Fig. 2). The demographic characteristics of the understudied patients are presented in Table 2.

**Table 2:** The demographic characteristics of the patients

Variable		Number	%
Gender	Man	9	30
	Woman	21	70
Age	Less than 55 years	10	33.3
	65 − 55 years	10	33.3
	More than 65 years	10	33.3
Education	High school and lower	25	83.3
	Diploma	3	10
	BSc degree and higher	2	6.7
Type of diabetes	Type 1 diabetes	6	20
	Type 2 diabetes	24	80
Duration diabetes	Less than 10 years	11	36.7
	More than 10 years	41	63.3
Type of GLM	Insulin	13	43
	Oral pills	12	40
	Insulin and pills	5	17
History of CVD	Yes	5	16
	No	25	84
History of proteinuria	Yes	7	23
	No	23	77

*GLM* glucose-lowering medicines, *CVD* Cardiovascular disease

**Fig. 3:**
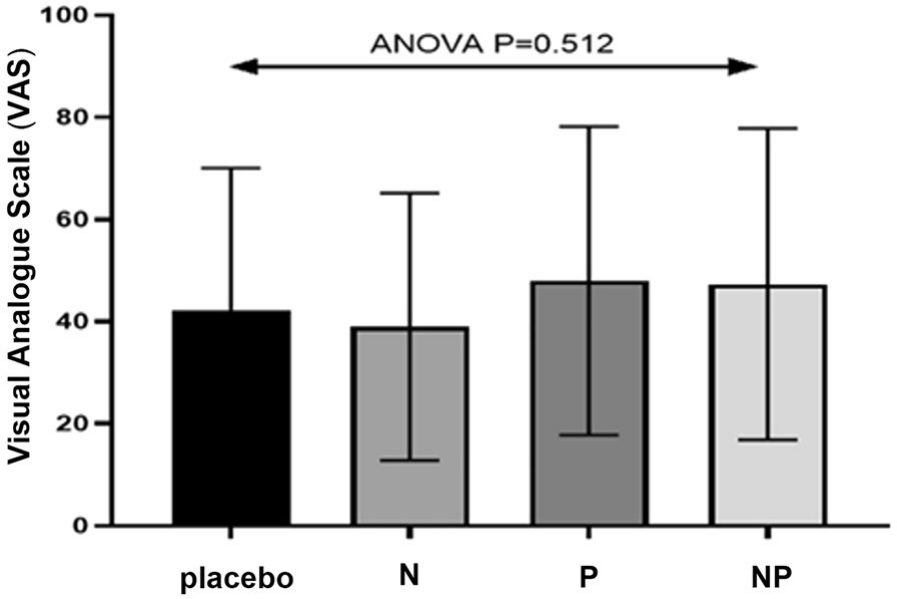
The mean VAS in the studied sessions, there was no significant difference in VAS levels between different sessions (P-value = 0.512). *N* a combination of analgesic drugs (including 325 mg acetaminophen, 200 mg ibuprofen, and 40 mg caffeine), *P* pregabalin (75 mg), *NP* a combination of N and P

**Fig. 4:**
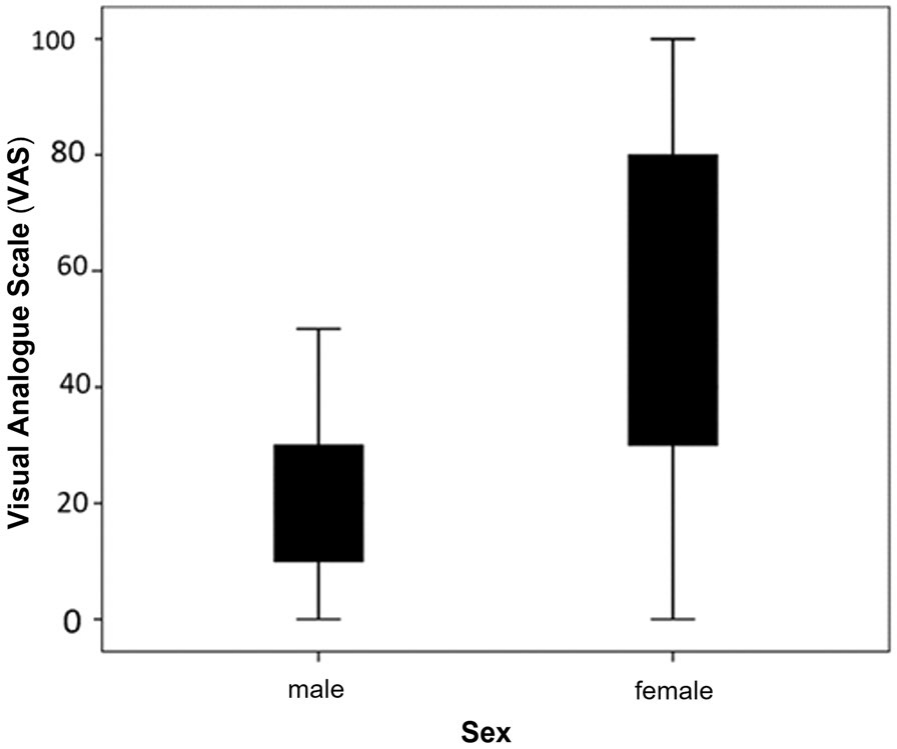
The mean VAS average level between males and females, the pain score was reported to be higher in women compared to that of men

The mean value of VAS at the placebo-received patients was 3.3 ± 1.8 units, while this scale was 3.1 ± 1.5, 3.5 ± 1.5, and 3.5 ± 1.8 in the N, P, and NP receiving groups, respectively. The VAS average in different sessions is shown in Fig. 3; no significant difference was observed in the VAS levels between different sessions (P = 0.512). Also, the VAS levels between each retinal quadrant were evaluated, but there were no significant differences between retinal quadrant sensitives regarding mean VAS scores. (P = O.642, Table 1). Different contextual variables, the order of drug administration in each session, and the clinical variables related to the surgery were analyzed in a univariate model to measure their impact on the pain. Among these variables, only sex had a significant effect on pain (P = 0.001). As shown in Fig. 4, the pain rate was reported to be higher in women than in men. By adjusting for sex, comparisons were made between pain rates in different groups by sex segregation and no significant difference was observed (P = 0.985 and 0.148, respectively).

Patients receiving P and the combination of NP showed a statistically significant decrease in IOP in their left eye after treatment. However, this decrease seems clinically non-significant. Moreover, in comparison between the four treatment sessions, systolic-diastolic pressure, pulse rate, and IOP in the right and left eye, no significant difference was observed before and after the procedure (Table 3).

**Table 3:** The measurements of systolic pressure, diastolic pressure, pulse rate, IOP right and IOP left in patients before and after receiving the drugs

	NP			P			N			placebo		
	Before	After	P	Before	After	P	Before	After	P	Before	After	P
SBP	142.43 ± 27.56	140.18 ± 7.27	0.653	140.90 ± 22.83	130.50 ± 22.83	0.277	141.67 ± 29.29	130.59 ± 84.87	0.418	145.23 ± 28.21	140.86 ± 85.37	0.344
DBP	87.67 ± 14.06	83.43 ± 11.74	0.792	83.97 ± 13.48	82.40 ± 10.20	0.029	82.47 ± 13.67	82.73 ± 10.92	0.032	84.00 ± 14.52	81.63 ± 11.22	0.304
Pulse	83.23 ± 12.35	79.17 ± 14.40	0.633	83.43 ± 12.99	83.40 ± 16.90	0.699	84.27 ± 10.17	71.20 ± 12.20	0.061	82.63 ± 10.47	91.70 ± 4.07	0.197
IOP R	16.13 ± 3.95	17.40 ± 4.79	0.012	16.90 ± 2.50	16.90 ± 4.38	0.633	12.34 ± 6.30	17.23 ± 2.70	0.023	17.03 ± 2.62	17.07 ± 4.09	0.471
IOP L	16.07 ± 3.80	14.67 ± 3.80	0.001	16.87 ± 2.67	14.13 ± 2.50	0.027	12.06 ± 6.53	14.20 ± 2.61	0.387	17.70 ± 3.11	14.86 ± 2.89	0.376

* N* a combination of analgesic drugs (including 325 mg acetaminophen, 200 mg ibuprofen, and 40 mg caffeine), *P* pregabalin (75 mg), *NP* a combination of N and P, *SBP* Systolic Blood Pressure, *DBP* Diastolic Blood Pressure, *R* Right, *L* Left

**Fig. 5:**
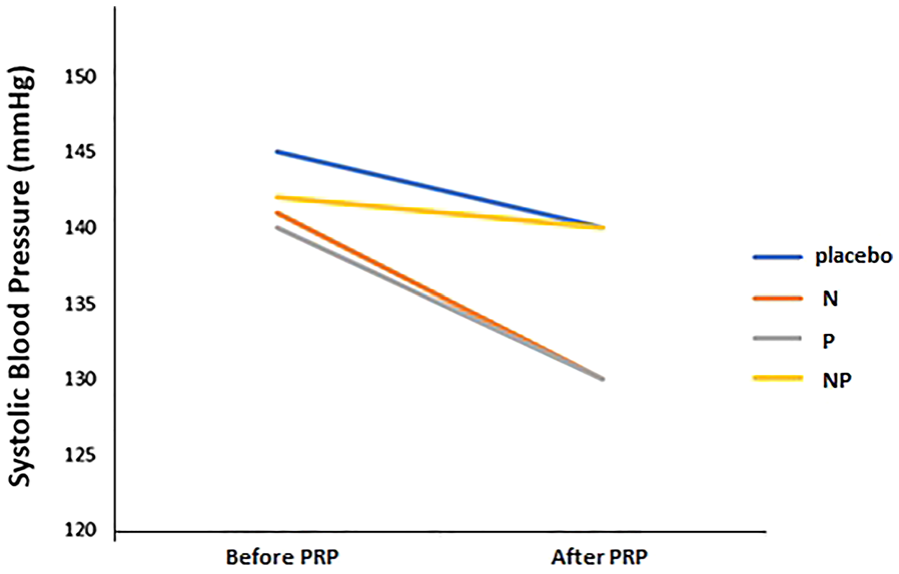
Comparison of systolic blood pressure changes in studied groups with adjustments for sex. *N* a combination of analgesic drugs (including 325 mg acetaminophen, 200 mg ibuprofen, and 40 mg caffeine), *P* pregabalin (75 mg), *NP* a combination of N and P

No significant difference was observed in the systolic pressure values in the four treatment sessions after adjusting for sex and age variables (multiple linear regression) (Fig. 5). The pulse rate and IOP changes also showed no significant alterations. No adverse effect was seen in any of the treatment sessions.

## Discussion

Despite the development of medical therapies for the treatment of PDR, PRP will likely continue as the mainstay of treatment. It is known that PRP is a painful procedure, which may lead to an insufficient remedy for the patient, increase the number of sessions, or perhaps the patient may even refuse to attend [10, 11]. The debate over the most effective method of pain control during PRP is persisting. Several analgesics were used to enhance patients’ compliance, from simple oral analgesics to the use of drugs applied in local anesthetic blocks and even, in some cases, general anesthesia [11].

Pain sensation and its quantification is a subjective index. In this study, we used VAS to evaluate the intensity of pain. The relationship between the pain-regulating systems and blood pressure suggests that acute pain during surgery can increase blood pressure and pulse rate by increasing sympathetic activity [12, 13]. Therefore, as objective indices, we used heart rate and systolic blood pressure to assess pain intensity during the procedure.

The present paper aimed to validate the analgesic effects of N and P capsules during the PRP procedure. There was no significant difference in pain relief between the sessions in which patient taking a placebo, N, P, or a combination of both during PRP.

Perioperative hemodynamic monitoring also showed no significant changes in the heart rate and systolic blood pressure during the time of using the drugs, before and after the PRP procedure. No significant drug related side effects were seen.

Wu et al. assessed the analgesic effects of acetaminophen and found non-effective results for pain control during PRP [6]. Ibuprofen is anNSAID medication that can relieve pain by inhibiting cyclooxygenase enzyme activity. In their clinical trial, Zakrzewski et al. evaluated diclofenac as an NSAID for reducing pain during PRP. They found that a single dose of oral diclofenac was an effective pretreatment analgesic agent for reducing pain experienced during PRP for PDR. However, this effect was not reported in their study or other studies on topical NSAIDs [14–16]. Contrary to their finding regarding the beneficial effect of the oral diclofenac, we did not find any significant analgesic effect when applying N capsules alone or in combination with pregabalin.

Pregabalin and its evolutionary predecessor, gabapentin, are structurally similar to gamma-aminobutyric acid (GABA), an inhibitory neurotransmitter, and can be used to manage diabetic neuropathic pain, postherpetic neuralgia, and fibromyalgia [17]. Few studies have been published on the efficacy of pregabalin during PRP.

Hazem et al. compared the analgesic efficacy and safety of oral gabapentin (600 mg) and pregabalin (150 mg) during PRP. They reported lower pain but more sedation and dizziness during PRP with preemptive pregabalin [18]. We applied pregabalin (75 mg) to avoid dizziness in the present trial. Still, contrary to Hazem et al. results, we did not find any positive effect in reducing pain caused by PRP when patients used pregabalin alone or in combination with the N capsules.

Of course, each person’s experience of pain and its expression is the product of the sensory experience; the individual’s background, cultural differences, and anxiety levels can influence this perception [19]. Also, a significant association between DR and diabetic neuropathy and their severities has been reported [20]. So, diabetic neuropathy may influence a patient’s pain sensation. To make minimally biased the result, we selected only patients with NPDR (intact retina) and also enrolled bilateral cases that had never experienced laser before. Finally, the same patient was submitted to PRP after either medication.

### Limitations

This study was associated with some limitations. First, the sample size was small. Second, although most of the laser parameters were not significantly different, the number of laser spots differed for each patient. Also, we did not perform the cognition tests before the procedure in this study which might be related to the patient’s VAS.

## Conclusion

This study found no evidence of the pain-relieving effect of N capsules (acetaminophen, ibuprofen, and caffeine), P capsules (pregabalin), or a combination of both medications during PRP. Further clinical studies must suggest the best drug regimen for pain relief during PRP in DR patients

## Data Availability

The data used to support the findings of this study are available from the corresponding author upon request.
